# DNA sequences within glioma-derived extracellular vesicles can cross the intact blood-brain barrier and be detected in peripheral blood of patients

**DOI:** 10.18632/oncotarget.13635

**Published:** 2016-11-26

**Authors:** Noemí García-Romero, Josefa Carrión-Navarro, Susana Esteban-Rubio, Elisa Lázaro-Ibáñez, María Peris-Celda, Marta M. Alonso, Juan Guzmán-De-Villoria, Carlos Fernández-Carballal, Ana Ortiz de Mendivil, Sara García-Duque, Carmen Escobedo-Lucea, Ricardo Prat-Acín, Cristóbal Belda-Iniesta, Angel Ayuso-Sacido

**Affiliations:** ^1^ Fundación de Investigación HM Hospitales, HM Hospitales, Madrid, Spain; ^2^ IMDEA Nanoscience, Madrid, Spain; ^3^ Facultad de Medicina (IMMA), Universidad San Pablo-CEU, Madrid, Spain; ^4^ Division of Pharmaceutical Biosciences, Faculty of Pharmacy, University of Helsinki, Helsinki, Finland; ^5^ Department of Neurological Surgery, University of Pittsburgh School of Medicine, Pittsburgh, PA, USA; ^6^ Clínica Universidad de Navarra, CIMA, Pamplona, Spain; ^7^ Servicio de Radiodiagnóstico, Hospital General Universitario Gregorio Marañón, Madrid, Spain; ^8^ Servicio de Neurocirugía, Hospital General Universitario Gregorio Marañón, Madrid, Spain; ^9^ Departamento de Neurocirugía, Hospital Universitario la Fe, Valencia, Spain

**Keywords:** extracellular vesicles, brain tumors, blood-brain barrier, biomarkers

## Abstract

Tumor-cell-secreted extracellular vesicles (EVs) can cross the disrupted blood-brain barrier (BBB) into the bloodstream. However, in certain gliomas, the BBB remains intact, which might limit EVs release. To evaluate the ability of tumor-derived EVs to cross the BBB, we used an orthotopic xenotransplant mouse model of human glioma-cancer stem cells featuring an intact BBB. We demonstrated that all types of tumor cells-derived EVs−apoptotic bodies, shedding microvesicles and exosomes−cross the intact BBB and can be detected in the peripheral blood, which provides a minimally invasive method for their detection compared to liquid biopsies obtained from cerebrospinal fluid (CSF). Furthermore, these EVs can be readily distinguished from total murine EVs, since they carry human-specific DNA sequences relevant for GBM biology. In a small cohort of glioma patients, we finally demonstrated that peripheral blood EVs cargo can be successfully used to detect the presence of IDH1^G395A^, an essential biomarker in the current management of human glioma

## INTRODUCTION

Primary malignant brain tumors account for 3% of adult cancer deaths and are the second cause of tumoral mortality in children [1]. High-grade gliomas are the most common primary malignant brain tumors in adults. Despite advances in treatment, the median patient survival rate is 12 to 15 months, as tumor eventually recurs in all patients [2].

Gliomas are commonly detected through clinical assessment and imaging techniques. However, the final diagnosis relies on the histological analysis of the biopsy tissue, in accordance with the WHO current standard for glioma diagnostic. Recently published molecular pathology-based glioma classification drastically improved tumor diagnostics and prognostics, essentially through the detection of the IDH1^R132H^ mutation [3, 4].

With the current treatment protocol, tissue specimens are suitable for the evaluation of tumor histopathology at the very beginning of the disease, but they do not allow molecular evolution assessment of the tumor along the course of the disease, which is critical for improving patient survival [5]. Thus, the last few years have seen a marked increase in using liquid biopsies for monitoring cancer genetics, by analyzing circulating cells, nucleic acids, or extracellular vesicles (EVs) released from tumors [6]. The EVs are present in readily accessible biofluids [7–9] and they carry lipids [10], proteins [11], and distinct species of nucleic acids [12–14] originating from donor cells. According to the Vesiclepedia nomenclature [15], three types of EVs can be distinguished: apoptotic bodies (ABs), shedding microvesicles (SMVs), and exosomes (EXOs). All of these EVs might be useful to identify the tumor molecular profile at any time, using minimally invasive procedures.

The amount and proportion of these circulating tumor-derived EVs might be determined by the integrity of the BBB, as well as the tumor size and distribution. Recent studies have demonstrated that EXOs administered intranasally [16] or injected through the tail vein in mice [17] can cross the BBB and deliver their cargo within the parenchymal brain, but the barrier's integrity was not discussed in these studies. Therefore, it remains unknown whether EVs of glioma cells can cross an intact BBB into blood vessels.

Tumoral mutated sequences have been recently detected in circulating DNA (ctDNA) and EVs extracted from cerebrospinal fluid (CSF) [18, 19]. However, CSF extraction through lumbar puncture is an invasive procedure and not recommended in patients with high intracraneal pressure (commonly present in brain tumors). In addition, bearing this in mind, additional efforts to develop a blood-based liquid biopsy method for GBM patients are required.

**Figure 1:**
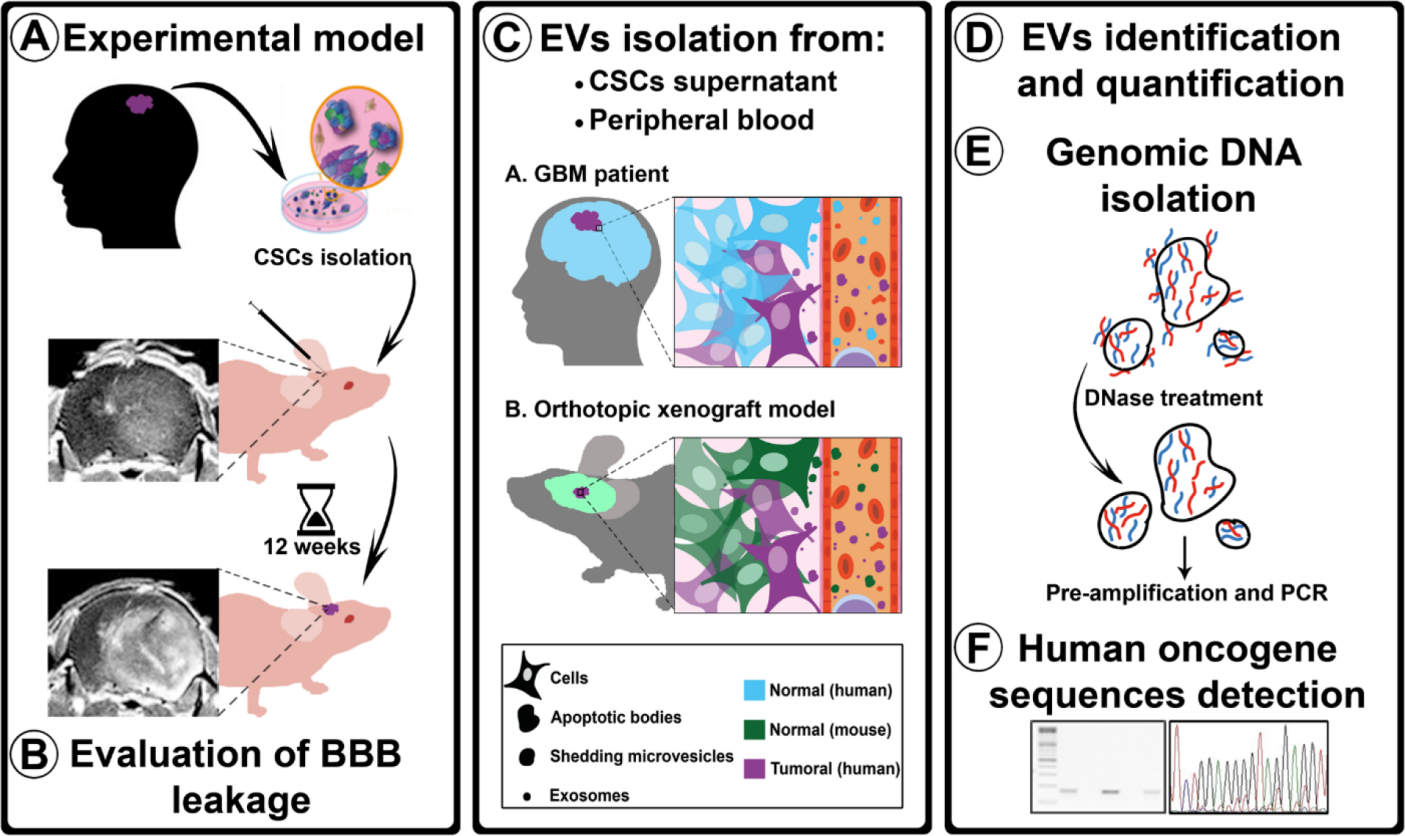
Experimental procedure flowchart **A.** Isolated hCSCs from 2 GBM patients were xenotransplanted in athymic mice. After 12 weeks, the animals were transcardially perfused. **B.** BBB permeability was evaluated using three assays: MRI, Evans Blue staining, and albumin extravasation. **C.** EVs (ABs, SMVs, and EXOs) were isolated from hCSCs-enriched culture supernatants and from mouse peripheral blood. **D.** EVs were identified using TEM, tracking analysis, and CD63 tetraspanin quantification. **E.** To ensure that the analyzed DNA was confined within the EVs, supernatants and plasma were treated with DNase before gDNA isolation; after the isolation, gDNA was pre-amplified before performing PCR analysis with human-specific primers. **F.** Sequences detected were sequenced to confirm their human origin.

To investigate the ability of tumor-derived EVs to cross an intact BBB, we used an orthotopic xenotransplant model of human cancer stem cells (hCSCs), previously described and characterized in our laboratory, which produces a disseminated brain-tumor phenotype featuring an intact BBB [20]. Using this model, we showed that all three types of EVs derived from human brain tumor cells can cross the undisrupted BBB and reach the bloodstream. These EVs carried human genomic-DNA (gDNA) sequences corresponding to those of the xenotransplanted cells, and could be isolated and enriched from peripheral blood (Figure 1). In a cohort of glioma patients with undisrupted BBB, we demonstrated that peripheral blood EV cargo can be successfully used to detect the presence of specific mutations, such as IDH1^G395A^. This finding provides evidence that liquid biopsies can successfully improve the current management of brain tumors.

## RESULTS

### GBM xenograft model with intact BBB

Our first aim was to evaluate the capacity of EVs secreted by glioma cells to cross normal BBB into the bloodstream. For this purpose, we used an orthotopic xenotransplant model of hCSCs culture (GBM27), which developed an infiltrative brain-tumor phenotype featuring an intact BBB in nude mice. We compared this model to a second orthotopic xenotransplant model of hCSCs culture (GBM38) that generated a nodular brain tumor featuring a disrupted BBB in nude mice (Supplementary Figure S1). We performed stereotactic transplants of 1 × 10^5^ cells from GBM27 and GBM38 into the striatum of mice. Twelve weeks later we evaluated the functional competence of the BBB through 3 distinct procedures (Supplementary and Figure S2).

**Figure 2:**
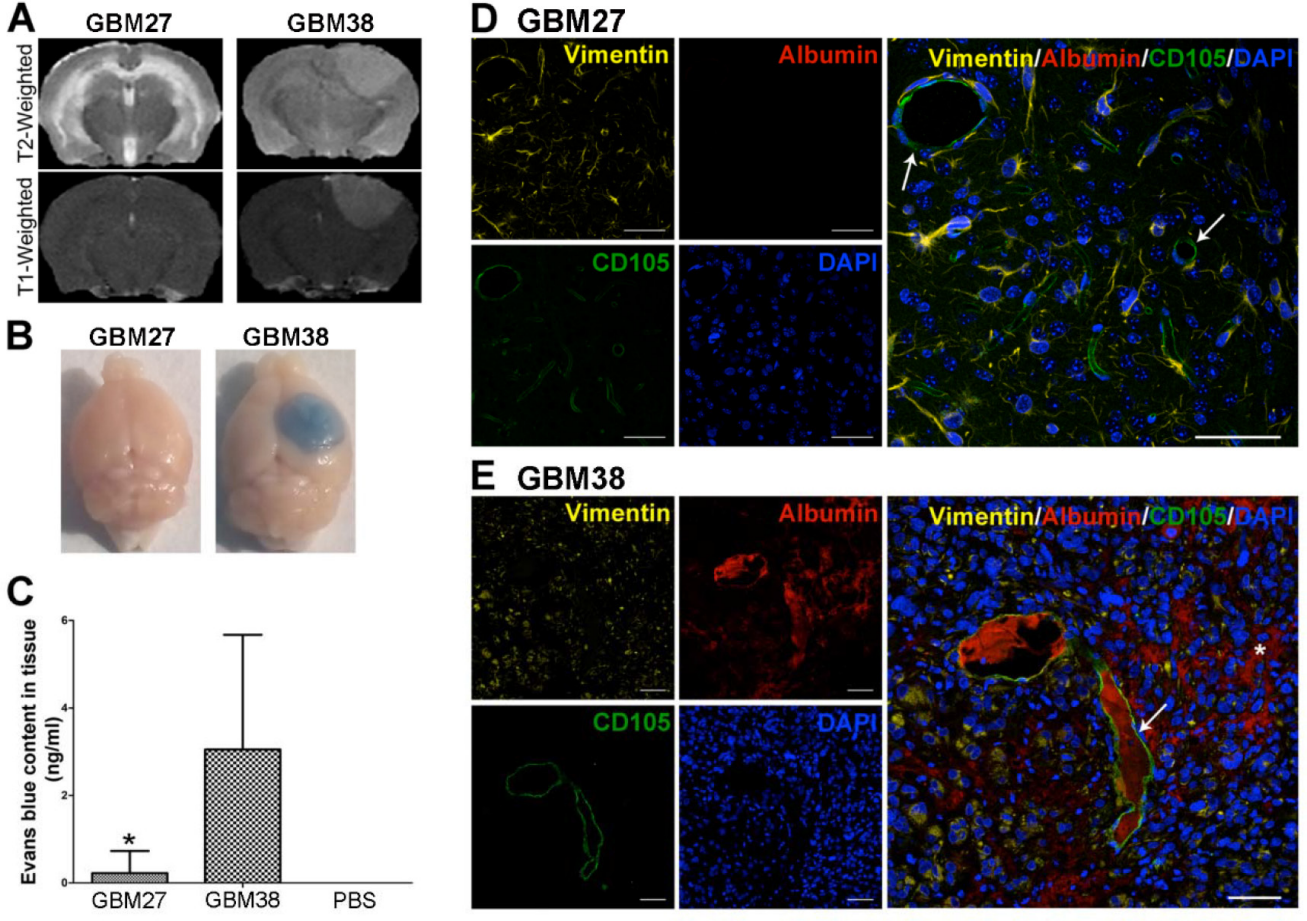
Evaluation of BBB leakage in two GBM models: GBM27 presents an intact BBB **A.** Representative T2- and T1-weighted images of GBM27 and GBM38. The GBM27 tumor xenotransplant T2-weighted image depicts diffuse hyperintense infiltrative involvement. GBM38 xenograft tumor showed well-defined borders. T2-weighted images revealed a hyperintense mass compressing ventricular structures. GBM27 features an intact BBB, as revealed by the lack of any contrast enhancement. GBM38 shows a homogeneous enhancement, suggesting that the BBB integrity is compromised. **B.** Evans Blue extravasation. Examination of the brains of perfused animals previously stained with Evans Blue confirmed BBB disruption in the GBM38 model. **C.** Quantification of Evans Blue extravasation. *P <0.05. **D-E.** Immunofluorescence staining of human vimentin (yellow), mouse CD105 (green), and mouse albumin (red). Nuclei were stained with DAPI (blue). GBM27 presents no sign of albumin staining throughout the tissue, which indicates that the BBB is intact. GBM38 features a leaky BBB, as shown by albumin spreading (white asterisk) from the blood vessels (white arrows) through the tissue. Total slides with anti-human vimentin are shown in Fig S1. Scale bar: 50 μm.

First, we evaluated the BBB integrity in the xenotransplant models GBM27 and GBM38 as well as in control mice (Figure 2A and Supplementary Figure S2A) with MRI images. On GBM38 xenograft tumor, we observed an enhancement in T1-weighted images with Gadolinium-based contrast agent, a sensitive marker of blood-brain barrier disruption (Gd-DTPA-BMA; Omniscan, Amersham Health, Oslo, Norway). Conversely, no enhancement of Gd-DTPA was observed on the GBM27 tumor suggesting an intact BBB. Control mice showed no MRI abnormalities.

To verify these findings, we visualised and quantified the amount of Evans Blue present in the parenchymal brain after its injection through the tail vein. This test showed that Evans Blue extravasation was significantly increased in the mice xenotransplanted with GBM38 cells compared to the GBM27 xenotransplanted ones (p < 0.05) (Figure 2BC), which confirmed that BBB was intact in GBM27 xenotransplants. No Evans Blue dye was detected in control brains (Supplementary Figure S2B). Finally, to demonstrate the functional competence of the BBB, we also analysed albumin extravasation using immunofluorescence (Figure 2D and E). Considerable albumin diffusion from blood vessels into the parenchymal brain of GBM38 xenotransplanted mice was observed (Figura 2D). Consistently, no albumin extravasation was detected in GBM27 xenotransplants nor in control mice (Figure 2 and Supplementary Figure S2D).

Our results confirmed that the GBM27 orthotopic xenotransplant model of hCSCs culture displays an intact BBB. This allowed us to examine whether tumor-derived EVs can cross intact BBB to the bloodstream.

### Morphological visualization and size distribution of EVs

**Figure 3:**
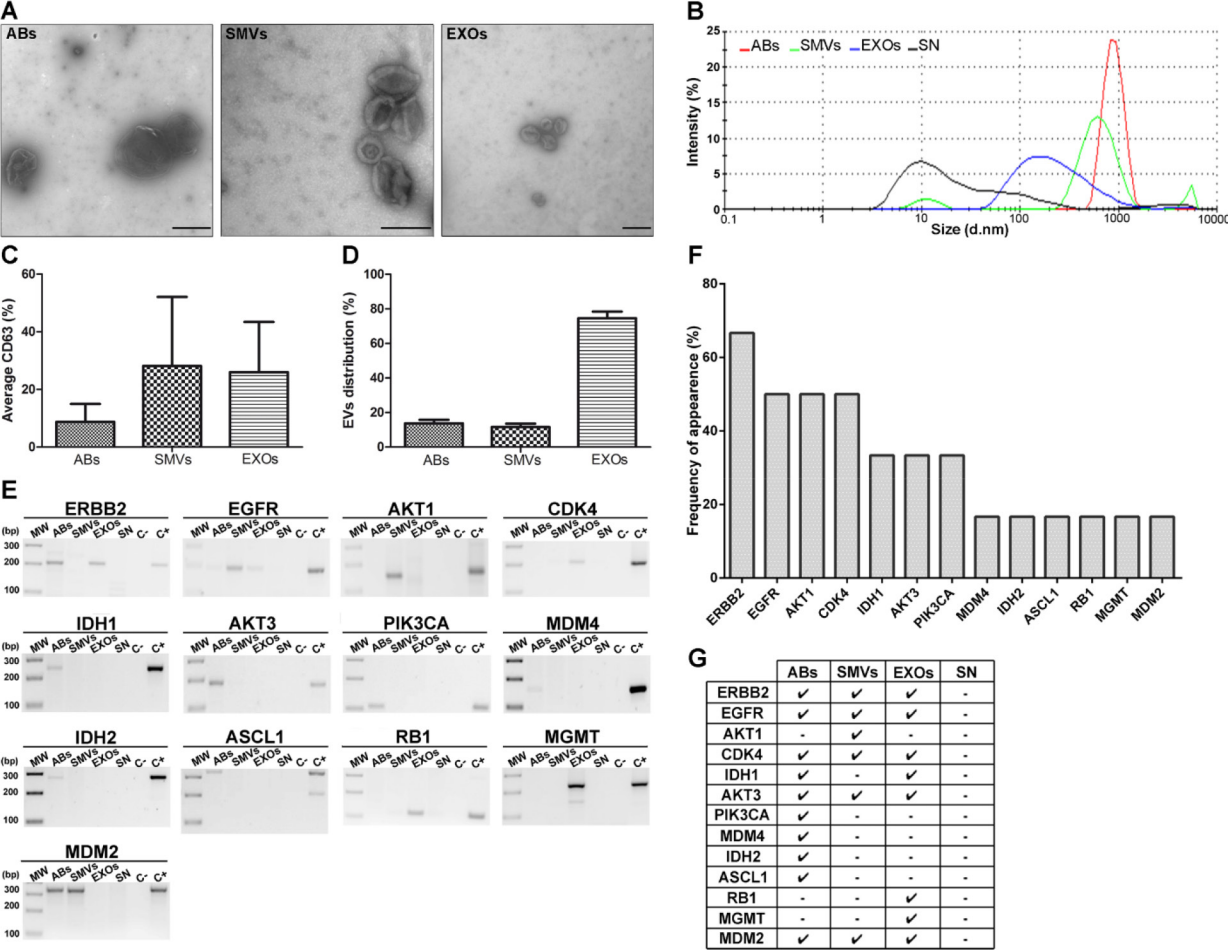
Morphological characterisation of EVs isolated from hCSCs supernatant from GBM27 and gDNA isolation **A.** Transmission electron microscopy images. ABs (500 nm to 1 μm), SMVs (500–150 nm) and EXOs (150–60 nm). **B.** Size distribution of EVs, as measured using Nanosizer tracking analysis. **C.** Quantification of the tetraspanin cell-surface glycoprotein CD63. **D.** Relative distribution of EVs. **E.** Most representative sequences analyzed are present in EVs isolated from GBM27 cells. **F.** Histogram showing the frequency of occurrence of target sequences after 6 consecutive experiments. **G.** Presence of *ERBB2*, *CDK4*, *AKT3*, and *MDM2* sequences in all types of EVs. The remaining sequences were found randomly in ABs, SMVs, and EXOs. No sequences were detected in the supernatant. Scale bars: 1 μm (ABs), 0.2 μm (SMVs and EXOs).

After demonstrating the suitability of our model, we studied whether all three types of EVs could be isolated and enriched from hCSCs (GBM27) culture supernatant as well as from plasma of xenotransplanted mice. EVs were isolated and enriched using centrifugation techniques, and then visualized using transmission electron microscopy (TEM) (Figure 3A and Supplementary Figure S3A). TEM images revealed the typical morphology and expected diameter ranges of the ABs (>1 μm), SMVs (~200 nm), and EXOs (~100 nm). To confirm the enrichment of the different fractions of EVs from GBM27 cells, we analyzed them using a Zetasizer: the AB fraction ranged from 2000 to 500 nm in size, the SMV fraction showed an average size of 600 nm, and the EXO fraction displayed a main peak at 180 nm (Figure 3B). The supernatant also showed a small 10-nm peak, compatible with the presence of proteins. These results confirmed the utility of the protocol used to isolate EVs from the supernatant. Although these images and sizes were consistent with the presence of all three types of EVs, we also quantified the relative expression of tetraspanin CD63. The results indicated similar CD63 expression in SMVs and EXOs and a considerably lower expression in ABs (Figure 3C). Lastly, EVs distribution was quantified using TEM: the EXO fraction was almost 4 times larger than the AB and SMV fractions in the hCSCs supernatant (Figure 3D). A similar trend was observed in the case of EVs isolated from mouse plasma (Supplementary Figure S3B).

### EVs from hCSCs contain gDNA

The presence of human gDNA in EVs was verified using a collection of primers specifically designed and validated to amplify human gDNA sequences (Supplementary Figure S4). We detected all the gDNA sequences assayed, except for *CDKN2a*, *PTEN*, and *TP53* sequences (Figure 3E). Then, we speculated whether the lack of PCR-amplification of these gene sequences might be related to the existence of chromosomal aberrations. Accordingly, we examined the chromosomal status of GBM27 cells using comparative genomic hybridisation (CGH). The CGH analysis revealed loss of heterozygosity of chromosome 9 at p21.3, chromosome 10 at q23.1 and chromosome 17 at position p13.1, which respectively affect the *CDKN2a*, *PTEN* and *TP53* locations (Supplementary Figure S5).

Interestingly, gDNA sequences relevant for the GBM biology were detected in all three types of EVs. Certain sequences appeared in all EVs, such as *ERBB2*, *EGFR*, *CDK4*, *AKT3*, and *MDM2*, whereas *IDH1* was detected only in ABs and EXOs, and a few sequences appeared exclusively in one type of EV: *PIK3CA*, *MDM4*, *IDH2*, and *ASCL1* in ABs, *AKT1* in SMVs, and *MGMT* and *RB1* in EXOs (Figure 3E-G). Nevertheless, we observed a 70,4% frequency of bias in this assay, which was independent of our experimental procedure (Supplementary Figure S6).

### hCSC-derived EVs cross the intact BBB into the bloodstream

**Figure 4:**
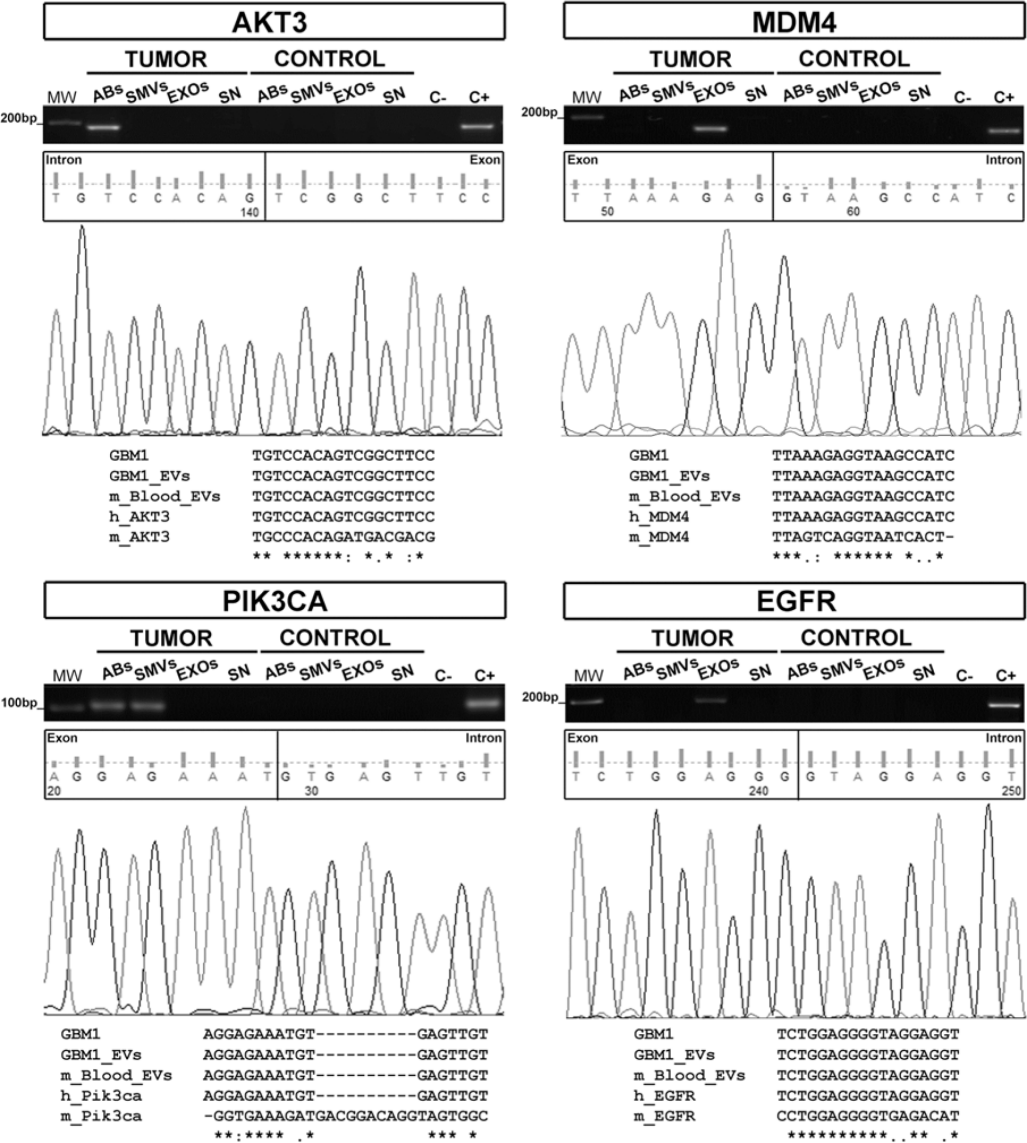
Human gDNA sequences are confined inside EVs isolated from xenografted mice *AKT3*, *MDM4*, *PIK3CA*, and *EGFR* sequences were detected in EVs isolated from the peripheral blood of 10 xenografted mice. The multiple sequence alignment shows complete homology among gDNA sequences from GBM27 hCSCs, EVs isolated from hCSCs-enriched culture supernatants, and EVs found in mouse peripheral blood. These results confirm the human origin of the sequences. Keyword: GBM1= GBM27.

The GBM27 orthotopic xenotransplant model was used to analyze whether all three types of GBM27-derived EVs can carry relevant human gDNA sequences, cross the undisrupted BBB and reach the bloodstream. We hypothesised that if this was the case, we should be able to detect relevant human gDNA sequences within the pool of EVs (both mouse and human) isolated from peripheral blood. Thus, we isolated and separated all three types of EVs from plasma obtained from the inferior vena cava, isolated total DNA from each type of EV and performed PCR assays to specifically amplify human gDNA sequences. PCR-amplification products of the expected sizes were obtained for *AKT3* from ABs, *PIK3CA* from ABs and SMVs, and *MDM4* and *EGFR* from EXOs. Multiple sequence alignment showed a complete homology among these sequences and their counterparts from the supernatants of hCSCs-enriched cultures (Figure 4).

### IDH1^G395A^ gDNA sequences successfully identified within EVs isolated from peripheral blood of human glioma patients regardless of BBB integrity

Having detected tumor-specific gDNA sequences within EVs isolated from peripheral blood in a mouse model with intact BBB, we next wondered whether glioma-derived EVs could be detected in the bloodstream of patients, regardless of the BBB integrity. To accomplish this, we screened peripheral blood EVs from 21 patients (20 diagnosed with low- and high-grade glioma and 1 brain metastasis) for the presence of IDH1 mutations, the most relevant mutation for human glioma diagnostic and prognostic [3] (Table 1). To investigate BBB integrity, we used the analysis of contrast acquisition in T1-weighted images, which takes advantage of the incapacity of gadolinium contrast agent to cross the intact BBB. In addition, we used Dynamic Contrast-Enhanced (DCE) MRI to longitudinally measure the vascular constant transfer K^trans^, which reflects BBB permeability (Supplementary Figure S7). The results revealed 3 patients (HGUGM002, HGUGM003 and HGUGM007) with an intact BBB, in contrast to the remaining 18 patients that showed a disrupted BBB (Table 1). Next, we assayed for the presence of IDH1^R132H^ on surgical solid samples by IHC (the Standard of Gold technique) and conventional PCR. IHC gave positive results for 3 low-grade (HGUGM003, HGUGM007 and HM001) and 1 high-grade (HGUGM008) glioma (Table 1). Consistent with these results, IDH1^G395A^ was also detected by conventional PCR on these 3 low-grade glioma samples. Furthermore, this technique detected IDH1^G395A^ in an additional low-grade glioma sample (HGUGM002), while it was negative for the high-grade glioma HGUGM008. Finally, we extracted total DNA from EVs from peripheral blood obtained immediately before surgery. As a first approach, we used conventional PCR to amplify IDH1 gDNA sequences, however, IDH1^G395A^ was observed in none of the low-grade gliomas, and only in 4 of the high-grade glioma samples, including HGUGM008, for which the presence of IDH1^R132H^ was detected on a surgical solid sample by IHC (Table 1). Considering these results, we hypothesized that IDH1^G395A^ sequences might be underrepresented within EVs compared to IDH1^wt^. To solve this problem, we tested the fast ColdPCR technique, which, in our hands, is able to enrich and detect IDH1^G395A^ when its relative representation is at least as low as 10% of total IDH1 sequences (Supplementary Figure S8). We used fast ColdPCR on solid samples, for which the results were similar to those obtained by conventional PCR. Notably, when we used fast ColdPCR on DNA isolated from peripheral blood EVs, we detected the presence of IDH1^G395A^ in 47.6% of the samples included in the cohort. Within the low-grade gliomas, IDH1^G395A^ was identified in 80% of the samples, matching our previous results from IHC and conventional PCR on solid samples. Interestingly, within high-grade gliomas, we also detected IDH1^G395A^ in 40% of the samples, including those previously detected by conventional PCR. The same technique identified only IDH1^wt^ on DNA isolated from peripheral blood EVs of a patient diagnosed with adenocarcinoma brain metastasis, used as a negative control.

**Table 1:** IDH1^G395A^ gDNA sequence identification in liquid biopsies from human glioma patients with or without BBB disruption

Patient ID	Patient variables		Pathological anatomy		BBB integrity evaluation by MRI-based techniques			Surgical sample analysis			Liquid biopsy analysis	
	Sex	Age	Pathologic diagnosis	Grade	BBB integrity	Contrast acquisition	Ktrans (10-3/min)	IHQ	FFPE Conventional PCR	FFPE Fast COLDPCR	EVs Conventional PCR	EVs Fast ColdPCR
**HGUGM002**	F	63	Oligodendro glioma	II	No disrupted	No	0	WT	G395A	G395A	N/D	G395A
**HGUGM003**	M	33	Oligodendro glioma	II	No disrupted	No	10	R132H	G395A	G395A	WT	G395A
**HGUGM007**	F	48	Oligoastro cytoma	II	No disrupted	No	N/D	R132H	G395A	G395A	N/D	G395A
**HM001**	M	31	Astrocytoma	II	Disrupted	Yes	N/D	R132H	G395A	G395A	WT	G395A
**HM012**	M	56	Astrocytoma	II	Disrupted	Yes	N/D	WT	WT	WT	N/D	WT
**HM009**	M	56	Astrocytoma	III	Disrupted	Yes	N/D	WT	WT	WT	WT	WT
**HGUGM004**	M	47	Grade III Astrocytoma with grade IV areas	IV	Disrupted	Yes	184-427	WT	WT	WT	N/D	WT
**HGUGM005**	F	43	Giant-Cells GBM	IV	Disrupted	Yes	N/D	WT	WT	WT	G395A	G395A
**HGUGM006**	M	53	GBM	IV	Disrupted	Yes	N/D	WT	WT	WT	WT	G395A
**HGUGM008**	M	39	GBM, recurrence	IV	Disrupted	Yes	N/D	R132H	WT	WT	G395A	G395A
**HGUGM009**	F	64	GBM, recurrence	IV	Disrupted	Yes	N/D	WT	WT	WT	N/D	WT
**HM002**	M	42	GBM	IV	Disrupted	Yes	N/D	WT	WT	WT	WT	WT
**HM003**	F	61	GBM	IV	Disrupted	Yes	N/D	WT	WT	WT	G395A	G395A
**HM004**	M	65	GBM	IV	Disrupted	Yes	N/D	WT	WT	WT	WT	WT
**HM005**	F	36	GBM	IV	Disrupted	Yes	N/D	WT	WT	WT	WT	WT
**HM006**	M	48	GBM	IV	Disrupted	Yes	N/D	WT	WT	WT	G395A	G395A
**HM007**	F	66	GBM	IV	Disrupted	Yes	N/D	WT	WT	WT	N/D	G395A
**HM008**	F	65	GBM	IV	Disrupted	Yes	N/D	WT	WT	WT	N/D	WT
**HM010**	M	43	GBM	IV	Disrupted	Yes	N/D	WT	WT	WT	N/D	WT
**HM011**	F	61	GBM	IV	Disrupted	Yes	N/D	WT	WT	WT	WT	WT
**HGUGM001**	F	61	Adenocarcinoma Brain metastases	O	Disrupted	Yes	638	WT	WT	WT	WT	WT

Samples are organized by tumor grade. BBB integrity is evaluated mainly by contrast acquisition, but also by K^trans^ when possible. Surgical tumor tissues were analyzed by IHQ, conventional PCR and Fast Cold PCR. Liquid biopsies were analyzed mainly by Fast Cold PCR. Keywords: BBB, blood brain barrier and N/D, not determined.

These results support the idea that EVs secreted by brain tumor cells can cross the BBB, whether intact or disrupted, and enters the bloodstream. Therefore, the analysis of their cargo might be useful as a biomarker not only for high-grade gliomas but also for low-grade gliomas, most of which conserve an intact BBB.

## DISCUSSION

The BBB maintains the brain environment and protects it against external factors. In glioma-diagnosed patients and xenografted mice, the BBB dysfunction is partially due to the impairment of tight junctions, which explains the fluid leakage and cerebral oedema associated with these tumors [21, 22]. However, a completely functional BBB is occasionally found in orthotopic xenograft mouse models [23], in certain low-grade human gliomas and a few high-grade gliomas [24]. In agreement with these reports, we demonstrated that the GBM27 orthotopic xenotransplant mouse model of hCSCs culture displays a functional BBB, using three approaches: Gd-DTPA MRI, external tracer Evans Blue, and evaluation of albumin extravasation by immunofluorescence. Thus, using the GBM27 model, we are able to assess the potential presence of brain tumor EVs in peripheral blood after crossing the intact BBB.

We have shown that gDNA sequences are present inside all three types of EVs isolated from the hCSCs supernatant. Other groups have also demonstrated the presence of gDNA sequences within total and fractioned EVs [13, 25].

Interestingly, we noted a key variability in the detection of specific gDNA sequences in the EVs, and based also on previous reports [26], we hypothesised that this variability might be more related to the representation of such sequences within the cell of origin than to the isolation and pre-amplification procedures. Although analysing the gDNA sequences contained within all three types of EVs might be useful for obtaining clinically relevant information, our results raise at least two technical issues. Firstly, the amount of tumor-derived EVs within the total pool of circulating EVs is relatively low. Secondly, the relative presence of mutated gDNA versus wild type sequences within tumor-derived EVs cargo is questionable. Further investigation is therefore required to elucidate the mechanisms underlying nucleic acid uptake by EVs.

Another critical factor that must be addressed in order to assess the use of EVs as biomarkers is the ability to separate EVs secreted by tumor cells from the total EVs pool. Here, we have shown that orthotopic xenotransplant animal models of human tumor cells are suitable models for providing ‘proof of concepts’ related to the utility of specific molecular biomarkers present within EVs.

The presence of circulating EVs secreted from glioma cells has been strongly correlated with tumor size and the BBB status [27]. Here, we demonstrated for the first time that human gDNA sequences relevant for the GBM biology can be detected within all three types of EVs isolated from the bloodstream of tumor-bearing mice. In this context, some groups have recently postulated the use of CSF as a source of tumor-derived DNA sequences [19, 21]. However, the lumbar puncture is often unfeasible in glioma patients mostly due to the high intracranial pressure [28].

Notably, our findings confirm that not only tumor-secreted EXOs but also ABs and SMVs can cross the intact BBB into the bloodstream. This is essential to standardize the use of circulating EVs in serum as glioma biomarkers regardless of the BBB status, providing a minimally invasive method compared to CSF. However, the exact mechanism used by EVs to cross the BBB remains elusive and might differ depending on the EV type.

Finally, the new molecular pathology-based classification of gliomas expanded current molecular knowledge about prognostic and diagnostic implications of a variety of biomarkers used to characterize glioma patients. For example, IDH1-mutated patients are expected to have longer survival rates than their wild-type counterparts despite similar histopathologic features [29]. Moreover, immunotherapy is a field of growing interest in brain oncology and specific glioma hallmarks are considered as immuno-targets. In this regard, a few IDH1 peptide vaccines trials are currently going on [30]. Unfortunately, there are obvious limitations to obtain a representative glioma tissue sample. This restrains the access to new therapies and clinical trials and impairs monitoring of drug resistance and/or clonal dynamics during treatment. As a consequence, almost all clinical trials accept first biopsies as a tissue to assess IDH1^R132H^ or any other target status. In this context, we addressed the potential use of tumor-derived EVs cargo in serum isolated from high or low grade brain tumor patients by looking for the presence of IDH1^G395A^, currently the most valuable molecular marker for human glioma diagnostics and prognostics [3]. In our hands, conventional PCR was useful to detect the wild type form, but unable to reveal the mutated form of IDH1 in DNA sequences from peripheral blood EVs, which might explain why previous works also reported negative results [19, 21]. Here, we demonstrated for the first time, that fast Cold-PCR can be successfully used to enrich the mutated form of IDH1 in DNA sequences from peripheral blood EVs isolated from brain tumor patients. In our cohort, the results obtained from liquid biopsies were consistent with those observed from solid samples of low-grade gliomas, but not with high-grade gliomas. The higher number of positive IDH1^G395A^ observed in high-grade gliomas, as compared with their corresponding solid samples, might be explained by the intra-tumor heterogeneity, for the small specimen analyzed by pathological anatomy barely represents the whole tumor. However, additional studies with larger cohorts are needed to consolidate and validate the use of DNA sequences isolated from the peripheral blood EVs of brain tumor patients.

In conclusion, we have demonstrated that all three types of EVs secreted by human glioma cells can cross the intact BBB. Furthermore, we prove that DNA sequences from peripheral blood EVs isolated from brain tumor patients can be successfully used to detect the presence or absence of specific molecular alterations such as IDH1^G395A^. This finding supports, for the first time, the utility of tumor-derived DNA within all three types of circulating EVs as potential biomarkers to improve diagnostics, prognostics and follow-up for both low- and high-grade gliomas.

## MATERIALS AND METHODS

### Human samples and derivation of glioblastoma-cancer stem cells-enriched cultures

Two tumor samples from GBM patients (GBM27 and GBM38) were processed within 12 h after extraction according to the protocol described previously [31]. Briefly, the samples were minced and washed in Ca^2+^/Mg^2+^-free HBSS (Hanks balanced salt solution). Enzymatic digestion was sequentially performed with Solution I (papain (14 U ml^−1^, Sigma-Aldrich) and DNase I (10 U ml^−1^, Sigma) in PIPES solution) for 90 min at 37°C and Solution II (papain (7 U ml^−1^) and DNase I (15 U ml^−1^) in 1:1 PIPES: proliferation medium) for 30 min at 37°C. The cells were then dissociated using diameter-tapering polished Pasteur pipettes, filtered through a 70-μm mesh, and resuspended in defined proliferative media. These CSCs lines were previously described and characterized in detail by our laboratory [20].

Solid surgical tissue samples and peripheralblood were obtained from patients operated at HM Hospitales (HM), Madrid, Spain; Hospital Universitario la Fe (HUlaFe), Valencia Sapin and Hospital General Universitario Gregorio Marañón (HGUGM), Madrid, Spain. Peripheralblood samples from patients were collected prior to surgery. These blood samples were left to clot for 30 min at room temperature and serum was isolated and stored at −80°C until use.

### Xenotransplants

Approximately 10^5^ cells from 2 cancer-stem cell cultures (GBM27 and GBM38) were injected into the right striatum of 10 immunosuppressed athymic nude female mice by using a stereotactic device. As a negative control, PBS was injected into 2 mice following the same protocol. Twelve weeks post-injection, mice were anaesthetised and then transcardially perfused with saline and prefixed with 4% paraformaldehyde. The brains were post-fixed for 48 h after the infusion and embedded in paraffin, after which 3-μm coronal sections were obtained.

### Magnetic resonance imaging (MRI) in mice

Magnetic resonance experiments were performed on a 7.0-T Bruker Pharmascan (Bruker Medical Gmbh, Ettlingen, Germany) superconducting magnet, with Paravision 5.1 software. T2-weighted MRI and T1-weighted MRI after paramagnetic contrast-agent administration were used to assess tumor implantation and BBB integrity, at time 0 and at 90 days. Prior to scanning, mice were anaesthetised with isofluorane.

T2-weighted images were acquired by using the rapid acquisition with refocused echo (RARE) sequence with the following parameters: TR/TE (repetition time/echo time) = 2500/44 ms, field of view = 2.3 cm, 6 averages, matrix size = 256 × 256, number of slices = 14, and slice thickness = 1 mm without a gap. The total scan time required to concurrently acquire T2-weighted images was 6 min.

Subsequently, we modified certain parameters for T1-weighted images; for example, we used the multi-slice multi-echo (MSME) sequence, TR/TE = 3500/10.6 ms, 3 averages, and total time of acquisition = 3 min 2 s. The contrast agent used, 0.3 M Gd-DTPA, was injected intraperitoneally.

### Evans blue injection and brain extraction

A 2% Evans Blue dye solution (Sigma-Aldrich) was administered (2 ml kg^−1^) through the tail vein and, 1 h later, the mice were transcardially perfused and the brains were extracted, weighed, and homogenised using a TissueLyser LT (Qiagen) in twice their volume of *N,N*-dimethylformamide (Sigma-Aldrich). The tissues were incubated overnight at 55°C and then centrifuged for 20 min at 9300 × *g*. The optical density (OD) of the supernatant was measured at 610–635 nm, and the amount of Evans Blue extravasation was quantified as nanograms per milligram of tissue.

### Immunofluorescence staining

Immunofluorescence staining for mouse albumin was performed to determine the vascular integrity of the brain. Brain sections obtained from GBM27 and GBM38 hCSCs and PBS control xenotransplants were incubated with a 5% blocking solution of the specific serum, and then incubated (overnight, 4°C) in solutions containing the following primary antibodies: goat anti-mouse CD105 (R&D Systems), goat anti-mouse albumin (Santa Cruz Biotechnology), and mouse anti-human vimentin (Santa Cruz Biotechnology). Then, Alexa Fluor-conjugated secondary antibodies were used for 1 h (donkey anti-goat 568, rabbit anti-goat 488, and goat anti-mouse 660; Life Technologies, USA), and then nuclei were counterstained with DAPI and coverslips were mounted using FluorSave™ reagent (Millipore). Fluorescence was examined under a Leica TCS SP5 inverted confocal microscope.

### Immunohistochemistry

Formalin-fixed paraffin-embedded sections were stained (as per the manufacturer's staining protocol) with the Bond Polymer Refine Detection Kit on a Bond-max™ fully automated staining system (Leica Microsystems GmbH, Germany), using a mouse monoclonal antibody against human IDH1^R132H^ (Clon H09, Dianova) for the detection of mutant IDH1^R132H^.

### Mice plasma samples

Twelve weeks after the xenotransplantation, mouse peripheral blood was collected from the inferior vena cava, and centrifuged at 1500 × *g* for 10 min to obtain the plasma.

### Extracellular vesicles isolation

EVs from plasma and serum samples and cell medium supernatants were isolated through differential ultracentrifugation as previously described [32], with certain modifications. Briefly, to remove cellular debris, samples were centrifuged at 1200 × *g* for 5 min. The supernatant was carefully aspirated off without disturbing the pellet and centrifuged at 8000 × *g* for 20 min to obtain ABs. Next, this supernatant was centrifuged at 25,000 × *g* for 20 min to obtain SMVs, and then the remaining supernatant was ultracentrifuged at 117,000 × *g* for 90 min to obtain EXOs (Optima-LE 80K ultracentrifuge, 50.2 Ti rotor; Beckman Coulter). All centrifugations were performed at 4°C. A sample of the EVs-free supernatant was collected after the ultracentrifugation and used as a negative control. In samples from human, total EVs were analyzed. Samples were immediately used or stored at −20°C.

### Transmission electron microscopy

EVs samples were individually added onto glow-discharged 150-mesh formvar copper grids (EMS™), subjected to the glow-discharge procedure (2 min, 2.4 MA), and then incubated for 2 min at 4°C. The grids were washed, negatively stained with 2% aqueous uranyl acetate solution, dried, and analyzed by performing TEM (FEI Tecnai Spirit G2 and Tecnai 12) at 80 kV. EVs were classified based on their size, photographed, and counted using a Soft Image System Morada camera.

### Tracking analysis

Mean droplet sizes of EVs were measured using the dynamic light scattering method and a Zetasizer Nano ZS (Malvern Instruments, UK).

### Flow-cytometry analysis of CD63 expression

AB, SMV, or EXO samples were adsorbed onto 4-mm aldehyde/sulphate latex beads (Invitrogen, Paisley, UK) overnight at 4°C. The reaction was stopped by adding glycine 100 mM. Membrane-bound beads were washed in PBS/1% BSA, incubated with mouse anti-CD63 (Abcam, Cambridge, UK) or appropriate isotype control for 30 min at 4°C, stained with FITC-conjugated secondary antibody (R&D Systems) for 30 min at 4°C, and resuspended in 0.5 ml of PBS. Samples were analyzed using a FACS Calibur flow cytometer (BD Biosciences, San Jose, CA, USA).

### DNA isolation and pre-amplification and PCR

Formalin-fixed paraffin-embedded (FFPE) samples were deparaffinized and extracted using the DNeasy Blood & Tissue Kit spin columns according to the manufacter's protocol. (Qiagen, Germany). Before DNA isolation, supernatant and plasma samples were treated with 27 Kunitz U ml^−1^ of DNase I (Qiagen) for 30 min at 37°C to remove potential free-DNA contaminants. Total DNA was extracted from EVs and cells by using a DNeasy Blood and Tissue Kit (Qiagen), as per manufacturer recommendations. Next, a working solution of the extracted DNA was pre-amplified using the GenomePlex Complete WGA2 Kit (Sigma-Aldrich), according to the manufacturer's instructions. All PCRs were performed following the protocol of the Paq5000 enzyme (Agilent Technologies) (Supplementary Figure S8B). The final PCR products were electrophoretically separated on 1.8% agarose gels.

### Fast Cold-PCR

For the enrichment and detection of IDH1 mutated gDNA sequences within EVs isolated from peripheral blood of human patients, DNA was amplified using the reported primers: forward 5′- CGGTCTTCAGAGAAGCCATT-3′ and reverse 5′-GCAAAATCACATTATTGCCAAC-3′ [33] (Supplementary Figure S8A). Cycling conditions were as follows: a first denaturation step of 10 min at 96°C, followed by a set of 20 cycles of 96°C for 15 sec and 60°C for 15 sec, and a second set of 30 cycles of 15 sec at T_c_ (critical temperature) and 60°C for 15 sec (Supplementary Figure S8C).

### Sequencing and assembly

DNA sequencing of the PCR-amplified products of *AKT3*, *EGFR*, *MDM4*, *PIK3CA* and *IDH1* was performed by the sequencing service at Spanish National Cancer Research Center (CNIO). Sequences were compared and aligned using the BLAST algorithm and CLUSTAL Omega, respectively.

### Dynamic contrast enhanced (DCE)-MRI data acquisition and analysis

Preoperative MR images were obtained using a 1.5 T MRI scanner (Achieva of Intera, Philips Healthcar, Best, The Netherlands) and 8-channel SENSE head coil. For DCE-MRI, baseline 3D T1-weighted images were obtained with the following parameters: TR 76 ms, TE 3ms, slice thickness 5mm, Field of View (FOV) 230 mm, matrix size of 116 x128, 35 volumes, temporal resolution 5,4 s and flip-angles of 5° and 15° to create two precontrast datasets. Then, a DCE perfusion imaging dynamic series was performed using T1-weigthed sequences with the same MR parameters except for an increased flip angle of 15°. At the end of the second volume acquisition, a bolus of 14 ml of gadobenate dimeglumine (Multihance, Bracco Imaging, Spain) was injected intravenously at a rate of 3-4 ml/s.

Structural contrast 3D T1 fast field echo (FFE) sequence was performed and the detail parameters were as follows: TR/TE= 4,6/9,4 ms, flip angle 8°, FOV 256 × 256 mm, matrix size 256x 256 and reconstructed voxel size of 1 × 1 × 1mm.

To observe BBB permeability, vascular constant transfer (K^trans^ min^−1^) values were calculated using Philips IntelliSpace Portal v.6 Software by simultaneous observation of axial postcontrast T1-weigthed images and corresponding colour parametric K^trans^ maps. One region of interest was manually positioned on the solid tumoral area.

### Statistics

Statistical analysis were performed using a 2-tailed Student *t* test. Data are presented as means ± standard deviation and were calculated using the software package GraphPad Prism v. 5.0. Statistical values of p > 0.05 were not considered significant.

### Study approval

Procedures used on mice were approved by and performed according to the guidelines of the institutional animal-care committee of the Principe Felipe Research Center in agreement with the European Union and National directives. Permission to use human samples was obtained from the ethical review board in HM Hospitales, Hospital Universitario la Fe, Valencia, and Hospital General Universitario Gregorio Marañón, Madrid and written informed consent was obtained from patients.

## SUPPLEMENTARY MATERIAL


