# Supplementary material for: DNA sequences within glioma-derived extracellular vesicles can cross the intact blood-brain barrier and be detected in peripheral blood of patients
Source: Oncotarget. 2016 Nov 26;8(1):1416–28. doi: 10.18632/oncotarget.13635 (PMC5352065; doi:10.18632/oncotarget.13635)
Supplement: Supplementary file 1 [file oncotarget-08-1416-s001.pdf]

DNA sequences within glioma-derived extracellular vesicles can cross the intact Blood-Brain Barrier and be detected in peripheral blood of patients

SUPPLEMENTARY MATERIAL

Human samples and derivation of glioblastoma-cancer stem cells-enriched cultures

Two tumor samples from GBM patients (GBM27 and GBM38) were processed within 12 h after extraction as per the protocol described previously [31]. Briefly, the samples were minced and washed in Ca^{2+}/Mg^{2+}-free HBSS (Hanks balanced salt solution). Enzymatic digestion was sequentially performed with Solution I (papain (14 U ml^{-1}, Sigma-Aldrich) and DNase I (10 U ml^{-1}, Sigma) in PIPES solution) for 90 min at 37°C and Solution II (papain (7 U ml^{-1}) and DNase I (15 U ml^{-1}) in 1:1 PIPES: proliferation medium) for 30 min at 37°C. The cells were then dissociated using diameter-tapering polished Pasteur pipettes, filtered through a 70-µm mesh, and resuspended in defined proliferative media.

Magnetic resonance imaging (MRI) in mice: T1- and T2- weighted images

T2-weighted images were acquired by using the rapid acquisition with refocused echo (RARE) sequence with the following parameters: TR/TE (repetition time/echo time) = 2500/44 ms, field of view = 2.3 cm, 6 averages, matrix size = 256 × 256, number of slices = 14, and slice thickness = 1 mm without a gap. The total scan time required to concurrently acquire T2-weighted images was 6 min.

Next, we modified certain parameters for T1-weighted images; for example, we used the multi-slice multi-echo (MSME) sequence, TR/TE = 3500/10.6 ms, 3 averages, and total time of acquisition = 3 min 2 s. The contrast agent used, 0.3 M Gd-DTPA, was injected intraperitoneally.

Dynamic contrast enhanced (DCE)-MRI data acquisition and analysis: T1-weighted images

For DCE-MRI, baseline 3D T1-weighted images were obtained with the following parameters: TR 76 ms, TE 3ms, slice thickness 5mm, Field of View (FOV) 230 mm, matrix size of 116 × 128, 35 volumes, temporal resolution 5,4 s and flip-angles of 5º and 15º to create two precontrast datasets. Then, a DCE perfusion imaging dynamic series was performed using T1-weigthed sequences with the same MR parameters except for an increased flip angle of 15º. At the end of the second volume adquisition, a bolus of 14 ml of gadobenate dimeglumine (Multihance, Bracco Imaging, Spain) was injected intravenously at a rate of 3-4 ml/s.

Structural contrast 3D T1 fast field echo (FFE) sequence was performed and the detail parameters were as follows: TR/TE= 4,6/9,4 ms, flip angle 8º, FOV 256 × 256 mm, matrix size 256x 256 and reconstructed voxel size of 1 × 1 × 1mm.

Immunohistochemistry

Formalin-fixed paraffin-embedded sections were stained (as per the manufacturer's staining protocol) with the Bond Polymer Refine Detection Kit on a Bond-max™ fully automated staining system (Leica Microsystems GmbH, Germany), using a mouse monoclonal antibody against human vimentin (V9) (Santa Cruz Biotechnology).

Primers design and PCR assays

Genomic sequences of known genes relevant for the GBM biology were obtained from the UCSC Genome Browser database and primers were designed covering intron/exon boundaries by using Primer 3 software. All primers were tested in PCR with human gDNA (positive control) and mouse gDNA (negative control). All PCRs were performed following the protocol of the Paq5000 enzyme (Agilent Technologies), and final PCR products were electrophoretically separated on 1.8% agarose gels.

Evaluation of DNA pre-amplification variability

To rule out the possibility that DNA pre-amplification procedures might be responsible for the variability observed in the PCR-amplification assays, we analysed the PCR-amplification results of 4 representative DNA sequences from hCSCs (*AKT3*, *MDM4*, *PIK3CA*, and *EGFR*) with or without the WGA kit. A representative gDNA sample from hCSCs was diluted to obtain a 1 ng μl^{-1} working solution and was pre-amplified using the GenomePlex Complete WGA2 Kit (Sigma-Aldrich). The same sample was also used without the pre-amplification step. *AKT3*, *MDM4*, *PIK3CA*, and *EGFR* were amplified using conventional PCR. Cycling conditions included an initial denaturation step of 2 min at 95°C, followed by 34 cycles of 20 s at 95°C, 20 s at the primer hybridisation temperature, and 30 s at 72°C, and a final extension step at 72°C for 5 min. The experiment was performed twice to ensure reproducibility of the result. We observed a

15.6% variability when the pre-amplification step was included; *AKT3* and *MDM4* showed 100% reproducibility, whereas certain amount of bias was observed for *PIK3CA* and *EGFR*. However, the absence of this step resulted in 100% reproducibility. Unexpectedly, we observed 70.4% variability when we used the gDNA isolated from EVs as a template. These results suggest that the high variability observed does not depend on the pre-amplification procedure, and instead depends mainly on the EVs cargo (Supplementary Figure S6).

SUPPLEMENTARY FIGURES

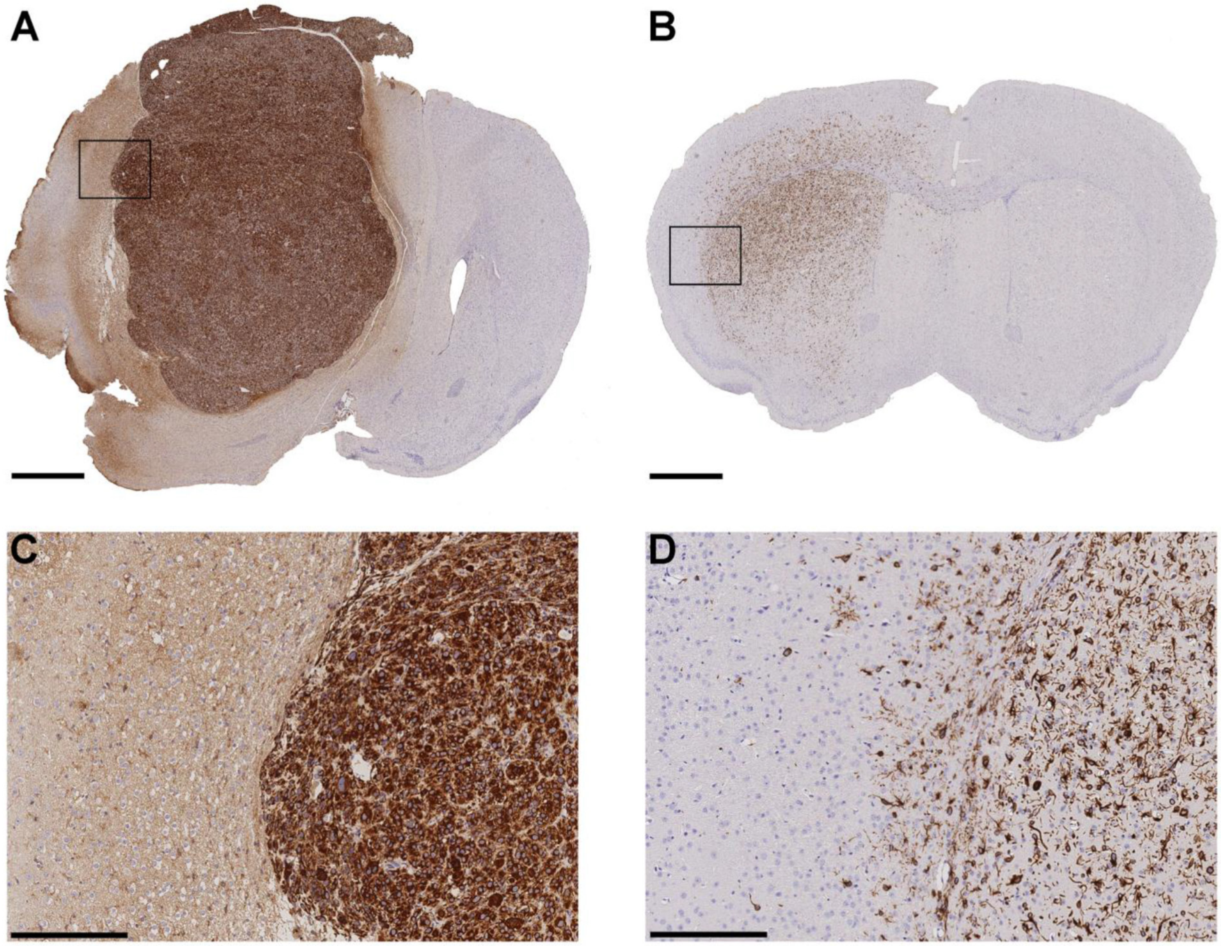

Supplementary Figure S1: Representative images of human vimentin staining. Histological analysis of paraffin-embedded tissue sections of formalin-fixed mouse brains. 3,3' Diaminobenzidine was used as the chromogen, and sections were counterstained with haematoxylin. **A.** GBM38 section showing a nodular growth pattern, with regular borders and no sign of invasiveness. **B.** GBM27 exibits diffuse growth, invading the tissue and migrating through the myelin tracts to the contralateral brain. Areas in the square are magnified in **C** and **D.** Scale bars: 1 mm (A, B), 200 µm (C, D).

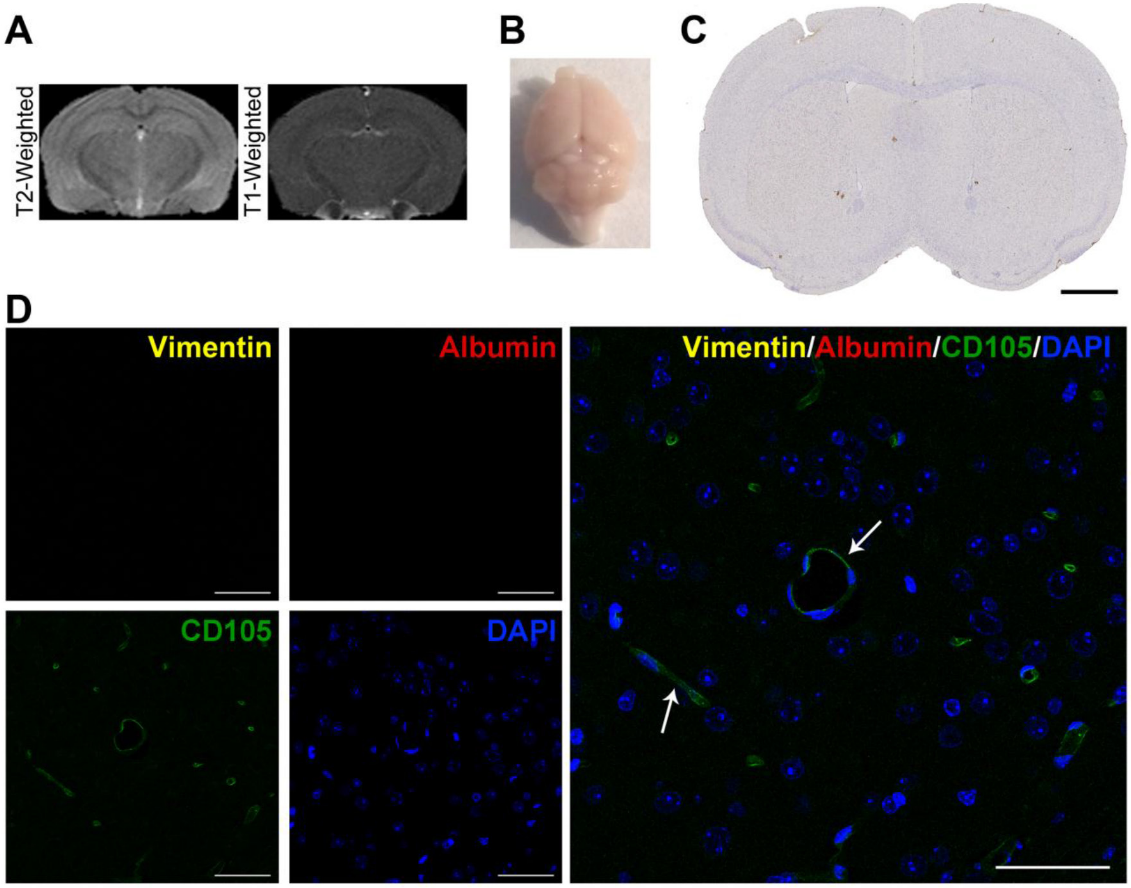

Supplementary Figure S2: Immunodeficient mice treated with control buffer (PBS) show an intact BBB. A. T2- and T1-weighted images of normal mouse brain injected with PBS. No abnormalities are shown. **B.** No Evans Blue dye extravasation. **C.** No human vimentin (V9) staining is detected, showing that no human cells were injected. **D.** Immunofluorescence staining, positive for mouse CD105 (white arrows) and negative for human vimentin (yellow) and mouse albumin (red). Nuclei were stained with DAPI (blue). Scale bars: 1 mm (C), 50 µm (D).

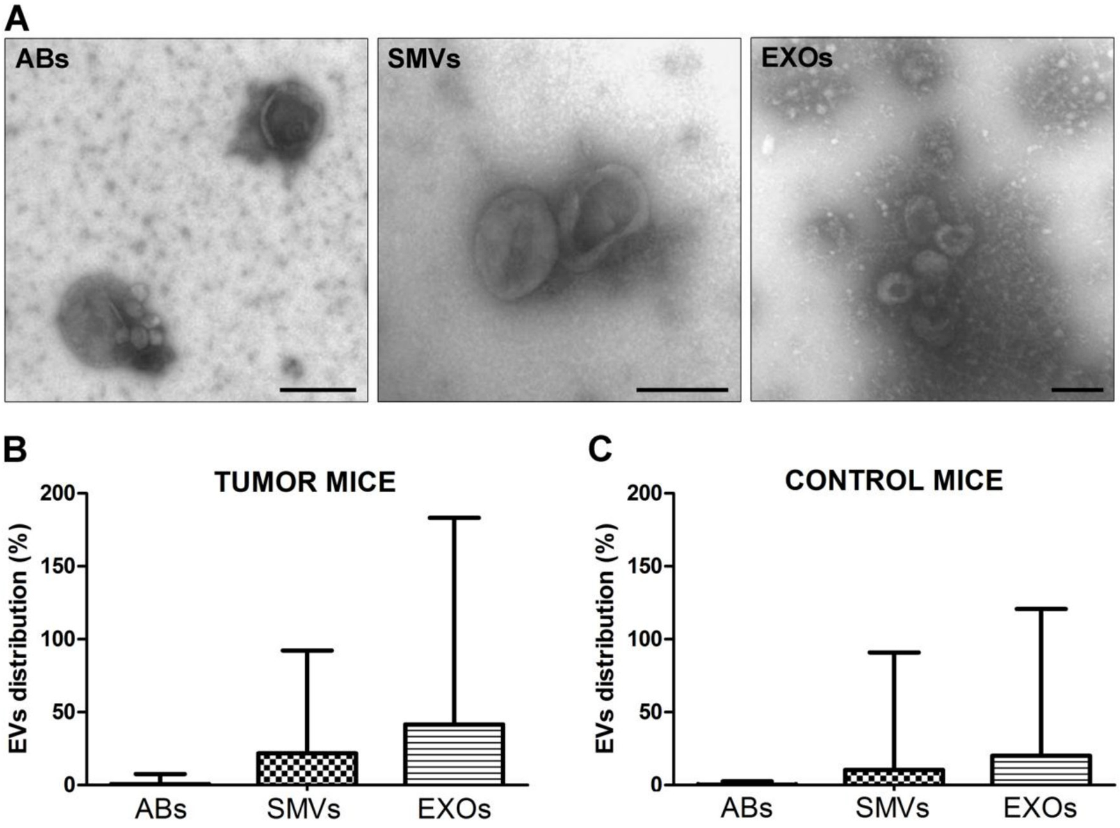

Supplementary Figure S3: Morphological characterisation and distribution of ABs, SMVs, ad EXOs in mouse plasma. **A.** Transmission electron microscopy images. Relative distribution of EVs in tumor **(B)** and control **(C)** mice. Scale bars: 0.5 µm (ABs), 0.2 µm (SMVs and EXOs).

GENE	PRIMER NAME	SEQUENCE	AMPLICON SIZE
TP53 NM_000546	TP53e7_F	CTTTGAGGTGCGTGTTTGTG	423
	TP53i8_R	caaccaggagccattgtctt	
PTEN NM_000314	PTENe2_F	GCAGAAAGACTTGAAGGCGTA	232
	PTENi2_R	aaatgaactgtatccccctgaa	
MDM2 NM_002392	MDM2i1_F	ctcagAATCATCGGACTCAGG	330
	MDM2i3_R	gctgtgtgaatgcgtcaaat	
MDM4 NM_002393	MDM4i2_F	atcagGTACGACCAAAACTGC	154
	MDM4i3_R	ctgggcaaaagagggagact	
AKT1 NM_001014431	AKT1i12_F	gagggtgtgctgggagtg	163
	AKT1e13_R	CTTGGTCAGGTGGTGTGATG	
AKT3 NM_005465	AKT3i13_F	gccgtggaaacactgaagtaa	183
	AKT3e14_R	GTAGGAAGCCGActgtggac	
CDK4 NM_000075	CDK4i2_F	gggttggtaggcattgagag	211
	CDK4e3_R	TCCACCACTTGTCACCAGAA	
CDKN2A NM_000077	CDKN2Ae2_F	GCACCAGAGGCAGTAACCAT	208
	CDKN2Ai2_R	agggcgatagggagactcag	
ERBB2 NM_004448	erbb2e25_F	CCCTTGGACAGCACCTTCTA	205
	erbb2i25_R	ggtaagcagacagccacaca	
EGFR NM_005228	EGFRi12_F	tgctgtgacccactctgtct	192
	EGFRi13_R	caacgcaaggggattaaaga	
ASCL1 NM_004316	ASCL1i1_F	cctccatctccctctaccac	320
	ASCL1e2_R	GACTCAGGTCCCAGTTGCTC	
MGMT NM_002412	MGMTi3_F	tgtgtagatgcgtttcctgttt	226
	MGMTe4_R	ATTGCTCCTCCCACTGCTC	
RB1 NM_000321	RB1e25_F	GGAAGCAACCCTCCTAAACC	111
	RB1i25_R	ccatctcggctactggaaaa	
PIK3CA NM_006218	PIK3CAe19_F	GGCTCAAAGACAAGAACAAAGG	103
	PIK3CAi19_R	ggaatacacaaacaccgacaga	
IDH1 NM_005896	Idh1i3_F	aggggaatgtctggacctct	245
	Idh1e4_R	TCCGTCACTTGGTGTGTAGG	
IDH2 NM_002168	IDH2e10_F	GAGAAGGTGTGCGTGGAGA	266
	IDH2i10_R	tcagggatggggaggaac	
G3PDH	hmG3PDH_F	ACACACAGTCCATGCCATCAC	450
	hmG3PDH_R	TCCACCACCCTGTTGCTGTA	

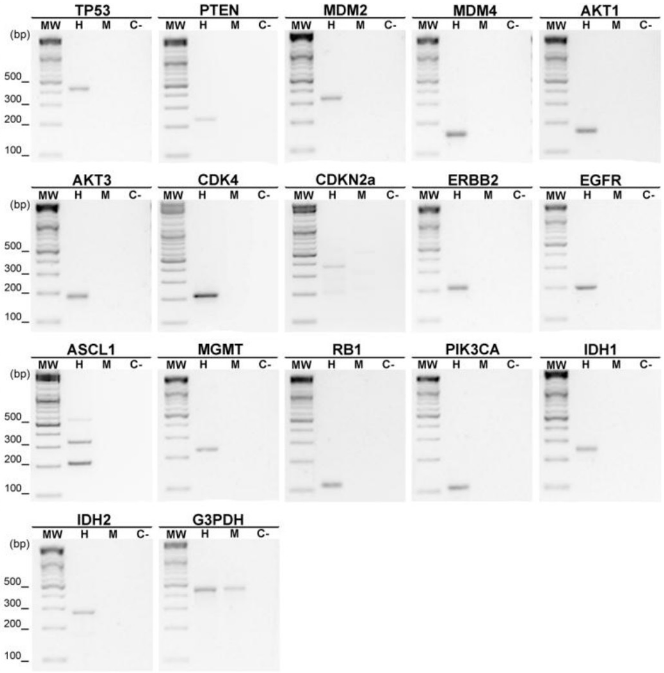

Supplementary Figure S4: Primer set designed using Primer 3 software and band size. Representative PCR products separated on a 1.8% agarose gel; the results show that all specific primers designed yielded amplified sequences of the expected size from human samples (H) but not mouse samples (M). *G3PDH* was the housekeeping gene used as reference in the human and mouse samples.

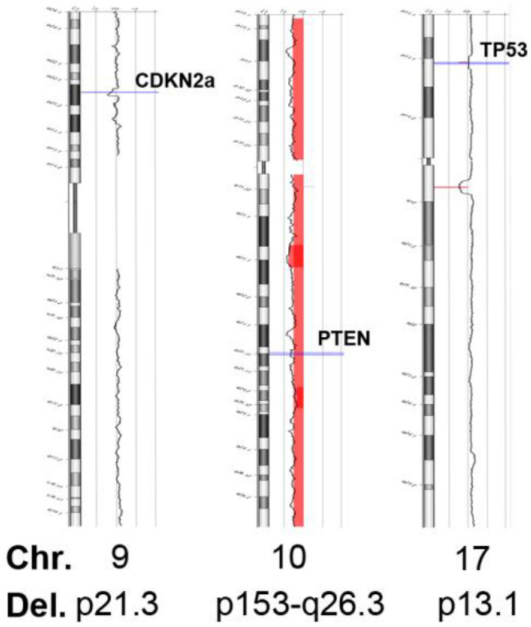

Supplementary Figure S5: Graphical representation of the Chromosomes 9, 10 and 17 of hCSCs culture (GBM27) by Comparative Genomic Hybridisation at passage 1. The figure shows loss of heterozygosity of chromosome 9 at p21.3, chromosome 10 at q23.1, and chromosome 17 at position p13.1, which respectively affect the *CDKN2a*, *PTEN*, and *TP53* locations.

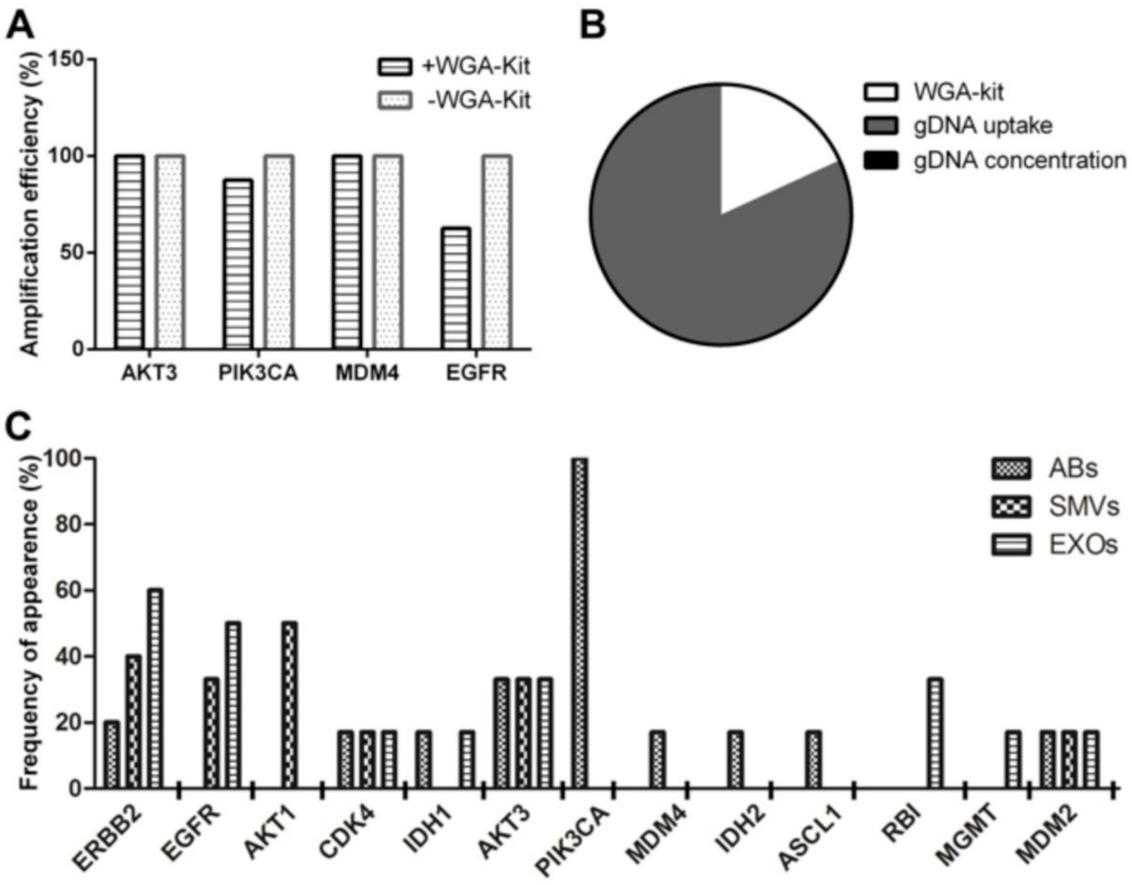

Supplementary Figure S6: Evaluation of DNA pre-amplification variability. A. Comparison of the variability induced with or without pre-amplification of 1 ng μl^{-1} of gDNA from hCSCs, based on PCR analysis of *EGFR*, *AKT3*, *PIK3CA*, and *MDM4* transcripts. **B.** Most of the bias observed appeared to be caused by gDNA uptake (70.4%), followed by the use of the WGA kit (15.6%). **C.** Detection frequency of analyzed sequences in each EVs fraction.

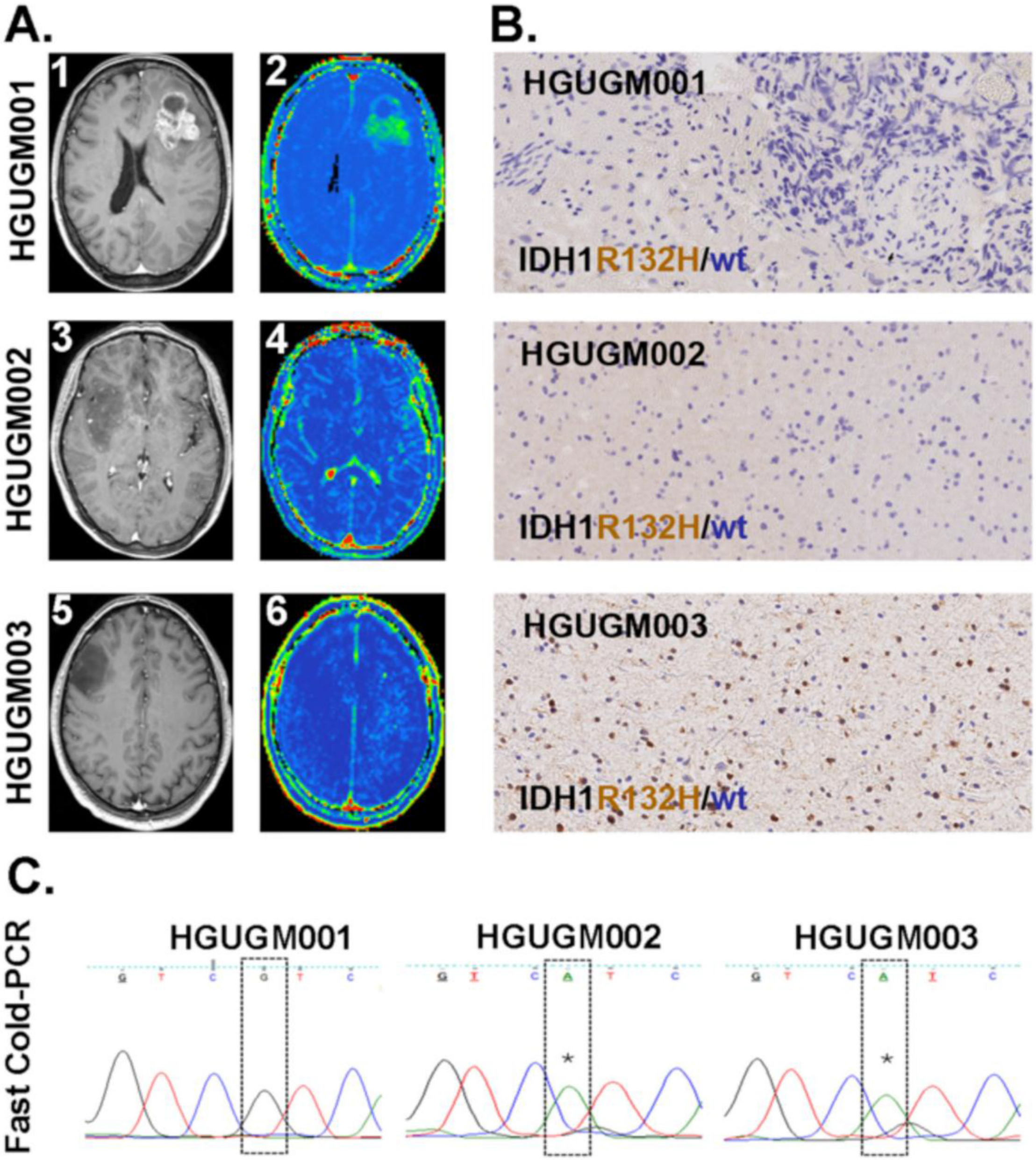

Supplementary Figure S7: Representative results showing the techniques used for the analysis of BBB state as well as the identification of IDH1^{G395A} on human glioma samples by immunohistochemistry and PCR. A. Post-contrast axial T1 weighted MRI of HGUGM001, HGUGM002 and HGUGM003 showing an adenocarcinoma brain metastasis (1) and non-enhancing low-grade gliomas (3 and 5) respectively. Representative K^{trans} maps from HGUGM001 (2), and HGUGM002 and HGUGM003 (4 and 6). Note that vasculature does not appear in these maps, and K^{trans} values in normal brain are insignificant. The K^{trans} values in the grade II tumor (4 and 6) are insignificant corresponding to the lack of enhancement with contrast. K^{trans} values are clearly elevated in peripheral brain metastasis. **B.** Representative IHC results (negative for HGUGM001 and HGUGM002 and positive for HGUGM003) on FFPE samples using a monoclonal antibody against human IDH1^{R132H}. **C.** DNA isolated from tumor-derived EVs extracted from peripheral blood and subjected to Fast ColdPCR. Fast ColdPCR rendered results consistent with those obtained on solid samples. * indicates the mutation G395A.

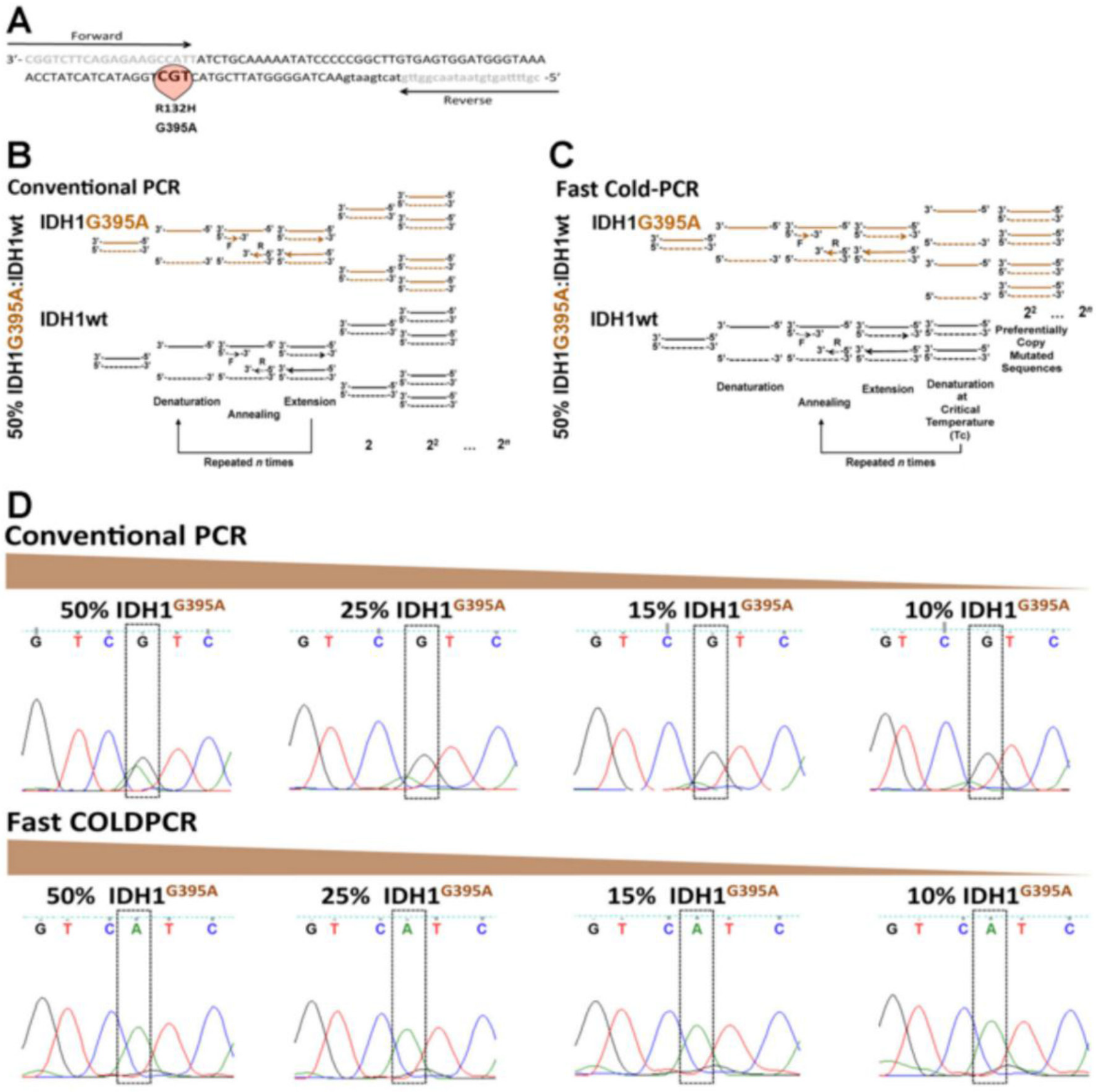

Supplementary Figure S8: Schematic representation of PCR procedures for the detection of IDH1^{G395A} and its identification on human glioma tumor samples. **A.** Specific primers designed to amplify out a 129 base pair amplicon flanking the 395 position within the human IDH1 genomic DNA sequence were used. For this aim two PCR amplification approaches were performed. **B.** Conventional PCR and **C.** Fast ColdPCR, which preferentially amplifies the mutated sequence. **D.** Conventional PCR was unable to detect and amplify IDH1^{G395A} when the presence of such a gDNA sequence appeared bellow a 50% of total IDH1 sequences. However, fast ColdPCR allowed for the selective enrichment and amplification of IDH1^{G395A} when its relative representation was at least as low as 10% of total IDH1 sequences.
